# Book Reviews

**Published:** 1898-09

**Authors:** 


Diseases of Women: A Clinical Guide to their Diagnosis and Treatment. By G. Ernest Herman, M. B., Lond., F. R. C. P., Obstetric Physician to and Lecturer on Midwifery at the London Hospital; Consulting Physician-Accoucheur to the Tower Hamlets Dispensary; Examiner in Midwifery to the Universities of London and Oxford, etc., etc. Octavo, pp. 886. Illustrated. New York: William Wood & Company. 1898.

It is a singular fact that, no matter how many treatises there may be on diseases of women, there always seems to be room for another, especially if it be written by a man with ample clinical experience, as is the case with the present author. Herman has upset the usual methods by writing of the different diseases, first according to their symptoms, instead of anatomically. This renders the book especially attractive to students and general practitioners of medicine, for whom it appears more especially to have been written.

The author's opening chapters are interesting, dealing as they do with pain, hysteria and headaches, but his methods of investigations are not as attractive as most teachers of recent writing. Especially is the chapter short and poorly illustrated, whereas to make a work of this kind appeal to would-be purchasers, the subject should be elaborately dealt with and profusely illustrated.

The work is divided into eleven parts and the foregoing remarks pertain to the principal features in the first section, which contains seven chapters. The second part, comprising seven chapters, is devoted to the consideration of chronic pelvic pain, which is one of the most important subjects in the whole range of gynecology, because it relates to the commoner ills for which women come daily to the consulting room. The noninflammatory conditions are here dealt with in a most practical way. In the next part pelvic inflammations are taken up, such as peritonitis, general or circumscribed, abscesses and the like. The author adheres to the old terms, perimetritis and parametritis—a nomenclature now somewhat obsolete in this country, but he makes his definitions so clear that there can be no mistaking the condition to which he applies these terms.

The fourth part, consisting of two chapters,treats of internal hemorrhage. In the first chapter, under the head of great internal hemorrhage, Herman deals with extrauterine pregnancy and for this as well as for all forms of severe bleeding he advises opening of the abdoman, in order to find the lesion and to deal with it surely and safely. This is the only sound teaching that can be offered on this subject. Hemorrhages of less severe nature are considered in the next part—the fifth—such as pertain to new growths, malignant or benign, and that relating to every pregnancy. This latter usually belongs to the department of obstetrics, but as many of the procedures for its relief are really gynecological there seems to be some reason for introducing it here. At all events it is admirably considered, showing the author to be well equipped in both branches.

In the next several parts the following-named conditions are respectively considered: in part six, leucorrhea; in part seven, disorders of the vulva; in part eight, disorders of menstruation; in part nine, disorders of the sexual functions; in part ten, disorders of parts adjacent to the sexual organs and in part eleven, abdominal tumors. All these subjects are clearly and ably handled. The author throws additional light upon some of these phases, and taken altogether the work is a most instructive and beautiful addition to the literature of gynecology.

The author's style is epigrammatic—sometimes dogmatic—but he writes because he has something to say and he stops after he has said it. The book is well printed, but its illustrations are not up to the present standard of American treatises on gynecology. The book is so practical that it must easily find its way to the library of progressive men and to the college list of text-books.

---

INTERNATIONAL CLINICS. A Quarterly of Clinical Lectures and Specially Prepared Articles on Treatment and Drugs. By professors and lecturers in the leading medical colleges of the United States, Germany, Austria, France, Great Britain and Canada. Edited by Judson Daland, M. D. (Univ. of Penna.), Philadelphia, Instructor in Clinical Medicine and Lecturer on Physical Diagnosis in the University of Pennsylvania; Assistant Physician to the Hospital of the University of Pennsylvania, etc.; Fellow of the College of Physicians of Philadelphia. J. Mitchell Bruce, M. D., F. R. C. P., London, England, Physician to and Lecturer on the Principles and Practice of Medicine at the Charing Cross Hospital. David W. Finlay, M. D., F. R. C. P., Aberdeen, Scotland, Professor of Practice of Medicine in the University of Aberdeen; Physician to and Lecturer on Clinical Medicine in the Aberdeen Royal Infirmary, etc. Volume II. Eighth series. 1898. Octavo, pp. xii.—366. Philadelphia: J. B. Lippincott Co. 1898.

The subjects considered in this number are drugs and remedial agents; treatment; medicine; neurology; surgery; gynecology and obstetrics; ophthalmology; laryngology; dermatology. One of the most interesting lectures is by James K. Young, of Philadelphia, on the Treatment of functional and lateral curvature of the spine. It is illustrated by eleven well-taken half-tones, demonstrating the author's mode of treatment. Another lecture of special interest is by J. F. Schamberg, of Philadelphia, entitled, Baldness, its varieties, causes and treatment. This lecture, too, is well illustrated and reflects credit upon its author. The other subjects average about as usual in this series, some being excellent and others mediocre. The book is better illustrated than common and makes a creditable addition to the series.

---

TRANSACTIONS OF THE SOUTHERN SURGICAL AND GYNECOLOGICAL SOCIETY. Tenth Annual Meeting, held at St. Louis, Mo., November 9, 10 and 11, 1897. William E. B. Davis, M. D., Secretary. Published by the Association. 1898.

True to its traditions this progressive association has again published an admirable book, full of instructive papers, that are amplified by strong and intelligent discussions. In these respects the association excels and the only wonder is that it does not reach its limit in the direction named. Each year, however, its transactions grow better and stronger and the present volume may be justly pronounced the best it has ever issued.

The president, Dr. George Ben Johnston, chose for the subject of his address The prevalence of specialism and who shall be specialists. It is needless to add that it received scholarly and forceful treatment at the hands of this accomplished physician. Then follows a series of papers embracing a wide range of surgical and gynecological subjects by some of the most prominent men throughout the South. The next president is Dr. Richard Douglas, of Nashville, and Memphis was selected as the place of meeting on the second Tuesday of November, 1898. The book is handsomely printed and bound, and the work of an experienced and tireless secretary is shown everywhere between its covers.

A Manual of Hygiene and Sanitation. By Seneca Egbert, A. M., M. D., Professor of Hygiene in the Medico-Chirurgical College of Philadelphia. In one 12mo volume of 360 pages, with 63 engravings. Philadelphia and New York: Lea Brothers & Co. 1898.

This author has constructed his work on different lines from those on which hygiene and sanitation are usually treated. After an introductory chapter he takes up in order bacteriology, the atmosphere, ventilation and heating, water, food, stimulants and beverages, personal hygiene, school hygiene, disinfection and quarantine, removal and disposal of sewage, vital statistics, and examination of air, water and food, devoting a chapter to the consideration of each.

As the author very properly asserts in his preface the science is too great and too important to be treated in a single volume, hence his aim has been to construct a book that students and others may use as an adjunct to further and more extensive reading.

Egbert has handled his subject in a scientific manner, but has not made it over-technical. A few illustrations serve to develop special points and its convenient size, as well as moderate price, commend it to undergraduate and postgraduate students.

---

SANITARY ENGINEERING. By WM. PAUL GERHARD, C. E., Consulting Engineer for Sanitary Works, Member of the American Public Health Association, etc. Duodecimo. New York: Published by the Author, 36 Union Square East. 1898.

This book deals with a comparatively new branch of civil engineering—one made necessary by the advent of that other comparatively new branch of preventive medicine, sanitary science. It is charmingly written and the subject is dealt with in a masterful manner. We wish that every person in the City of Buffalo who has to do in any way with the cleaning of our streets from the mayor down to the inspectors was obliged to read carefully what this author says under the head of street cleaning. One sentence deserves to be pondered by the street railway officials. It reads as follows: "Street railroad companies, after using the snow plow to clear their tracks, should by city ordinance be required to remove that portion which they pile up outside the tracks." What say you to that, Mr. Watson? We have nothing but praise for this little brochure and it should be in the hands of every city official as well as every sanitarian.

---

TRANSACTIONS OF THE NINETEENTH ANNUAL MEETING OF THE AMERICAN LARYNGOLOGICAL ASSOCIATION, held at Washington, D. C., May 4, 5 and 6, 1897. HENRY L. SWAIN, M. D., Secretary. New York: D. Appleton & Company. 1898.

The American Laryngological Association is one of the oldest and best of our national special societies and its annual volume of transactions always contains the most modern exposition of laryngological science. The present book deals with the technique of removing nasal growths and correcting deformities in an intelligent manner, several authors having presented papers relating to these subjects. Again, nasal and throat disease amenable to treatment, apart from surgery, are also set forth with skill; especially is laryngeal phthisis an important disease of this class and its management always becomes a matter of great interest. Dr. Charles H. Knight, of New York, was president at this meeting and his knowledge and skill in this department of medicine made itself felt in the excellence of the papers and good discussions. Every laryngologist should possess these annual volumes.

ATLAS OF METHODS OF CLINICAL INVESTIGATION, with an Epitomé of Clinical Diagnosis and of Special Pathology and Treatment of Internal Diseases. By DR. CHRISTFRIED JAKOB, of Erlangen. Edited by AUGUSTUS A. ESHNER, M. D., Professor of Clinical Medicine in the Philadelphia Polyclinic, etc. Duodecimo, 259. Illustrated. Philadelphia: W. B. Saunders. 1898.

This is one of the most helpful handbooks that has lately been sent out to the profession. The plates are exceptionally good, those relating to the blood ranking on the superb order. Everything that contributes to accuracy in diagnosis should be welcomed by the general practitioner of medicine and in this book he will find much to aid him in the direction named. The drawings are of a nature to even teach more than the text, but the latter is all-sufficient to elaborate the former. We commend the book to student and practitioner.

